# Supplementary material for: Evaluation of association studies and a systematic review and meta-analysis of CYP1A1 T3801C and A2455G polymorphisms in breast cancer risk
Source: PLoS One. 2021 Apr 28;16(4):e0249632. doi: 10.1371/journal.pone.0249632 (PMC8081265; doi:10.1371/journal.pone.0249632)
Supplement: S2 Table — (PDF) [file pone.0249632.s002.pdf]

Supplemental Table 2. Genotype distribution of CYP1A1 polymorphisms in the included studies of BC (HB hospital-based study, PB population-based study, FB family-based study)

No.	First Author/Year	Country	Ethnicity	Sample size	Source of control	Type of control	Matching	T3801C genotype distribution						HWE	Quality score	A2455G genotype distribution						HWE	Quality score
								Case			Control					Case			Control				
								TT	TC	CC	TT	TC	CC			AA	AG	GG	AA	AG	GG		
1	Hayashi S [1] 1992	Japan	Asian	98/358	PB	Healthy controls	ND	–	–	–	–	–	–	–	–	65	29	4	233	108	17	0.332	12
2	Ambrosone CB [2] 1995	USA	Caucasian	176/228	PB	ND	Age	–	–	–	–	–	–	–	–	140	32	4	195	31	2	0.539	14
3	Bailey LR [4] 1998	USA	Caucasian	164/162	HB	Non-cancer patients	Age	130	30	4	136	21	5	0.001	12	149	15	0	150	12	0	0.625	12
4	Bailey LR [4] 1998	USA	African	59/59	HB	Non-cancer patients	Age	42	15	2	33	23	3	0.692	11	58	1	0	59	0	0	<0.001	11
5	Fontana X [5] 1998	France	Caucasian	487/100	Volunteers	Healthy women	ND	363	121	2	77	21	2	0.689	11	–	–	–	–	–	–	–	–
6	Ishibe N [6] 1998	USA	Mixed	466/464	PB	ND	Age	379	87		385	79		–	13	405	61		370	64		–	13
7	Huang CS [7] 1999	China	Asian	141/145	HB	Healthy women	ND	49	60	32	48	80	17	0.06	12	71	64	8	80	53	12	0.449	12
8	Taioli E [8] 1999	USA	African	21/85	PB	Healthy women	ND	7	10	4	51	31	3	0.513	13	20	0	0	78	5	0	0.777	13
9	Taioli E [8] 1999	USA	Caucasian	30/183	PB	Healthy women	ND	22	8	0	146	32	5	0.06	14	24	5	0	145	28	2	0.625	14
10	Moysich KB [10] 1999	USA	Mixed	154/192	PB	ND	Age	–	–	–	–	–	–	–	–	127	27		168	23		–	14
11	Basham VM [11] 2001	UK	Caucasian	1948/1355	PB	Non-cancer controls	ND	–	–	–	–	–	–	–	–	1818	126	4	1250	104	1	0.438	16
12	Krajinovic M [12] 2001	Canada	Caucasian	135/200	PB	Healthy women	Age	120	14	1	180	19	1	0.524	12	130	5	0	183	22	0	0.417	14
13	Dialyna IA [13] 2001	Greek	Caucasian	207/171	HB	Healthy women	ND	156	48	3	123	45	3	0.629	10	–	–	–	–	–	–	–	–
14	Miyoshi Y [14] 2002	Japan	Asian	195/272	Volunteers	Healthy women	ND	85	83	27	86	139	47	0.473	11	131	52	12	156	94	22	0.15	11
15	Laden F [15] 2002	USA	Mixed	367/367	PB	Non-cancer women	Age	294	73		299	68		–	16	316	51		311	56		–	16
16	Wu FY [16] 2002	China	Asian	60/60	HB	Non-cancer women	Age	50		10	49		11	–	8	–	–	–	–	–	–	–	–
17	da Fonte de Amorim L [17] 2002	Brazil	Caucasian	79/123	HB	Non-cancer women	Age	55	24		83	40		–	9	–	–	–	–	–	–	–	–
18	da Fonte de Amorim L [17] 2002	Brazil	Mixed	49/133	HB	Non-cancer women	Age	33	16		67	66		–	8	–	–	–	–	–	–	–	–
19	Miyoshi Y [18] 2003	Japan	Asian	257/191	PB	Healthy women	ND	120	137		74	117		–	13	–	–	–	–	–	–	–	–
20	Zhu J [19] 2003	USA	Mixed	45/100	HB	Non-cancer controls	ND	74	26		31	14		–	9	–	–	–	–	–	–	–	–
21	Hefler LA [20] 2004	German	Caucasian	391/1699	HB	Healthy women	ND	332	56	3	1361	325	13	0.179	11	361	28	1	1570	117	6	0.019	11
22	Zhang Y [21] 2004	USA	Caucasian	374/406	PB+HB	Non-cancer controls	Age	279	95		324	82		–	15.5	334	40		385	21		–	15.5
23	Li Y [22] 2004	USA	Caucasian	413/415	PB	ND	Age	327	78	8	325	83	7	0.526	14	385	29	3	378	37	2	0.299	14
24	Li Y [22] 2004	USA	African	265/280	PB	ND	Age	155	93	17	165	102	13	0.179	14	259	12	0	274	11	0	0.74	14
25	Boyapati SM [24] 2005	China	Asian	1120/1196	PB	Non-cancer controls	Age	433	517	170	453	556	187	0.45	19	659	421	51	694	442	73	0.814	19
26	Le Marchand L [25] 2005	USA	Mixed	1339/1370	PB	ND	ND	743	493	103	722	530	118	0.143	14	–	–	–	–	–	–	–	–
27	Okobia M [27] 2005	Nigeria	African	220/218	HB	Non-cancer patients	Age	135	69	16	130	71	17	0.107	9	–	–	–	–	–	–	–	–
28	Modugno F [28] 2005	USA	Caucasian	249/3954	PB	Non-cancer controls	ND	–	–	–	–	–	–	–	–	234	15		3676	278		0.41	16
29	Chacko P [29] 2005	India	Indian	112/112	HB	Non-cancer controls	Age	62	39	11	90	18	4	0.022	10	67	34	11	90	19	3	0.126	10
30	Chang TW [30] 2006	China	Asian	189/421	HB	Healthy women	Age	71	118		134	287		–	12	118	70		233	188		–	12
31	Gallicchio L [31] 2006	USA	Caucasian	86/1318	PB	Non-cancer patients	ND	–	–	–	–	–	–	–	–	78	7	1	1233	80	5	0.004	17
32	Li JY [32] 2006	China	Asian	89/136	HB	Healthy women	ND	–	–	–	–	–	–	–	–	26	45	18	49	66	21	0.874	13
33	Shen Y [33] 2006	China	Asian	250/268	PB	Non-cancer women	Age	83	125	42	128	109	31	0.295	17	–	–	–	–	–	–	–	–
34	Shin A [35] 2007	Korea	Asian	380/286	HB	Non-cancer controls	ND	147	173	60	95	118	73	0.004	12	252	213	28	232	175	30	0.698	12
35	Sillanpää P [36] 2007	Finland	Caucasian	481/479	PB	Healthy women	ND	–	–	–	–	–	–	–	–	426	53	2	412	66	1	0.327	16
36	Singh N [37] 2007	India	Indian	146/162	PB	Normal volunteers	ND	94	35	17	94	53	15	0.072	10	98	36	11	119	41	2	0.46	10
37	Guo WD [38] 2007	China	Asian	144/154	HB	Healthy women	ND	47	92	25	65	73	18	0.714	11	–	–	–	–	–	–	–	–
38	Torresan C [40] 2008	Brazil	Mixed	102/102	Volunteers	Non-cancer controls	Age	74	28		81	21		Y	10	–	–	–	–	–	–	–	–
39	Diergaarde B [41] 2008	USA	Mixed	319/637	PB	Non-cancer controls	Age	258	61		495	142		–	15	291	26		584	49		–	16
40	Li JY [42] 2008	China	Asian	78/78	HB	Healthy women	Age	18	60		33	45		–	9	–	–	–	–	–	–	–	–
41	Gulyaeva LF [43] 2008	Russia	Caucasian	118/188	ND	Non-cancer women	ND	74	34	10	148	40	0	0.103	3	–	–	–	–	–	–	–	–
42	Ociepa-Zawal M [44] 2009	Poland	Caucasian	71/100	HB	Non-cancer patients	ND	65	6	0	82	18	0	0.323	7	63	7	0	87	13	0	0.487	8
43	Sangrajrang S [45] 2009	Thailand	Asian	568/496	HB	Healthy women	ND	148	303	117	127	248	121	0.997	15	282	215	47	242	201	37	0.594	15
44	Surekha D [46] 2009	India	Indian	248/249	Volunteers	Healthy women	Age	–	–	–	–	–	–	–	–	78	169	1	122	127	0	0	12
45	Shimada N [47] 2009	Japan	Asian	403/403	HB	Non-cancer controls	Age	175	173	55	168	187	48	0.713	15	246	137	20	249	134	20	0.72	15
46	Shimada N [47] 2009	Brazil	Asian	81/81	HB	Non-cancer patients	Age	31	40	10	37	36	8	0.86	12	46	30	0	50	28	3	0.704	13
47	Shimada N [47] 2009	Brazil	Mixed	389/389	HB	Non-cancer patients	Age	255	115	19	252	120	17	0.574	14	300	78	9	314	70	5	0.626	15
48	Kato I [48] 2009	USA	African	194/189	PB	Women controls	Age	117	68	9	109	67	13	0.541	14								
49	Moreno-Galván M [49] 2010	Mexico	Mixed	91/94	HB	Healthy women	ND	21	51	19	35	50	9	0.141	11	29	48	14	38	41	15	0.485	11
50	MARIE-GENICA [50] 2010	German	Caucasian	3148/5482	PB	Non-cancer controls	Age	2624	510	14	4538	902	42	0.7	18	2934	210	2	5099	378	7	0.998	18
51	Syamala VS [51] 2010	India	Indian	359/367	HB	Non-cancer women	ND	148	143	68	243	106	18	0.154	11	–	–	–	–	–	–	–	–
52	Cui Z [52] 2010	China	Asian	315/360	PB	Non-cancer controls	ND	138	120	57	214	114	32	0.005	13	–	–	–	–	–	–	–	–
53	Kiruthiga PV [53] 2011	India	Indian	50/50	HB	Non-cancer controls	ND	17	30	3	12	36	2	<0.001	7	36	2	12	50	0	0	<0.001	7
54	Wang Q [54] 2011	China	Asian	400/400	HB	Healthy women	Age	–	–	–	–	–	–	–	–	215	149	36	224	152	24	0.79	16
55	dos Santos RA [55] 2011	Brazil	Mixed	62/62	ND	Non-cancer women	ND	40	22		53	9		–	5	–	–	–	–	–	–	–	–
56	Tuerxun M [56] 2011	China	Asian	130/148	HB	Healthy women	ND	54	60	16	79	62	7	0.235	10	–	–	–	–	–	–	–	–
57	Chen J [57] 2011	China	Asian	135/112	HB	Healthy controls	ND	35	78	22	39	61	21	0.732	11	–	–	–	–	–	–	–	–
58	Naushad SM [58] 2011	India	Indian	342/253	HB	Healthy controls	ND	168	125	49	140	98	15	0.692	12	221	108	13	144	99	10	0.163	12
59	Ashley-Martin J [59] 2012	Canada	Caucasian	204/619	PB	Non-cancer controls	Age	160	44		465	154		–	15	–	–	–	–	–	–	–	–
60	Reding KW [60] 2012	USA	Caucasian	1133/982	PB	Non-cancer controls	ND	918	199	16	764	202	16	0.531	18	1056	72	7	896	83	2	0.957	18
61	Reding KW [60] 2012	USA	African	472/444	PB	Non-cancer controls	ND	269	186	17	263	156	25	0.768	17	461	18	0	431	13	1	0.012	17
62	Khvostova EP [61] 2012	Russia	Caucasian	276/274	HB	Healthy women	ND	179	88	9	218	55	1	0.203	9	–	–	–	–	–	–	–	–
63	Huang Y [62] 2013	China	Asian	144/152	HB	Healthy women	ND	78	55	11	59	79	14	0.086	11	–	–	–	–	–	–	–	–
64	Wang H [63] 2013	China	Asian	80/60	HB	Healthy women	ND	21	46	13	16	33	11	0.404	9	–	–	–	–	–	–	–	–
65	Martínez-Ramírez OC [64] 2013	Mexico	Mixed	150/150	PB	Healthy women	Age	–	–	–	–	–	–	–	–	39	37	74	57	43	50	0	10
66	Petchkovskiy EV [65] 2014	Russia	Caucasian	670/480	HB	ND	ND	–	–	–	–	–	–	–	–	564	54	3	388	32	0	0.417	12
67	Saadatian H [66] 2014	Iran	Caucasian	100/100	HB	Non-cancer women	ND	–	–	–	–	–	–	–	–	82	17	1	93	7	0	0.717	10
68	Ghisari M [67] 2014	Danish	Caucasian	30/113	ND	ND	Age	–	–	–	–	–	–	–	–	4	17	9	32	54	27	0.652	11
69	Zhang M [68] 2014	China	Asian	286/310	HB	Healthy controls	ND	85	119	82	108	134	68	0.033	12	–	–	–	–	–	–	–	–
70	Oliveira CB [70] 2015	Brazil	Mixed	742/742	BD	Healthy women	Age	464	236	42	456	243	43	0.165	15	527	195	20	570	157	15	0.283	15
71	Tuerxun M [71] 2015	China	Asian	144/120	HB	Healthy controls	ND	62	63	19	65	49	6	0.399	11	–	–	–	–	–	–	–	–
72	Amrani I [73] 2016	Jordan	Caucasian	112/115	HB	Non-cancer controls	Age	–	–	–	–	–	–	–	–	102	10	0	102	12	1	0.349	12
73	Farzaneh F [74] 2016	Iran	Caucasian	124/100	HB	Healthy controls	Age	34	69	21	30	56	14	0.135	13	–	–	–	–	–	–	–	–
74	Pazilat Abdurixit [75] 2016	China	Asian	200/200	HB	Healthy controls	ND	73	96	31	94	87	19	0.861	12	–	–	–	–	–	–	–	–
75	García-Martínez A [76] 2017	Mexico	Mixed	923/978	PB	Healthy controls	Age	288	450	185	307	484	187	0.878	16	368	409	150	338	488	161	0.495	16
76	Ghisari M [77] 2017	Danish	Caucasian	142/196	CR	Non-cancer women	Age	–	–	–	–	–	–	–	–	138	4	0	187	8	1	0.012	17
77	Parada H [78] 2017	USA	Caucasian	937/985	PB	Non-cancer women	Age	–	–	–	–	–	–	–	–	873	60	4	890	92	3	0.705	17
78	Liang FQ [79] 2018	China	Asian	337/469	HB	Healthy women	Age	117	159	61	152	243	74	0.155	13	–	–	–	–	–	–	–	–
79	Qiu J [80] 2018	China	Asian	427/536	Volunteers	Non-cancer women	Age	136	216	75	173	280	83	0.082	16	–	–	–	–	–	–	–	–
80	Naif HM [81] 2018	Iraq	Caucasian	199/160	ND	Non-cancer women	ND	32	59	108	66	64	30	0.047	8	40	62	97	74	65	21	0.269	8
81	Khalili-Tanha G [82] 2019	Iran	Caucasian	96/110	PB	Healthy controls	Age	–	–	–	–	–	–	–	–	75	21	0	91	17	2	0.271	12

HB: hospital-based, PB: population-based, CR: cancer registry, ND: not described, BD: Blood donors
