# Supplementary material for: Evaluation of association studies and a systematic review and meta-analysis of CYP1A1 T3801C and A2455G polymorphisms in breast cancer risk
Source: PLoS One. 2021 Apr 28;16(4):e0249632. doi: 10.1371/journal.pone.0249632 (PMC8081265; doi:10.1371/journal.pone.0249632)
Supplement: S3 Table — (PDF) [file pone.0249632.s003.pdf]

S3 Table Genotype frequencies of the CYP1A1 polymorphisms between and breast cancer and control groups by menopausal status

First author/Year	Ethnicity	CYP1A1 T3801C genotype distribution						CYP1A1 A2455G genotype distribution					
		Case			Control			Case			Control		
		TT	TC	CC	TT	TC	CC	AA	AG	GG	AA	AG	GG
Postmenopausal													
Amrani [73] 2016	Asian	–	–	–	–	–	–	33	1		34	2	
Ghisari [67] 2014	Caucasian	–	–	–	–	–	–	2	12		15	56	
Martínez-Ramírez [64] 2013	Mixed	–	–	–	–	–	–	19	18	31	26	17	26
dos Santos [55] 2011	Mixed	24	8		17	6		–	–	–	–	–	–
Wang [54] 2011	Asian	–	–	–	–	–	–	95	65	16	100	68	8
Moreno-Galván [49] 2010	Mixed	13	36	15	20	26	4	19	32	13	25	19	6
MARIE-GENICA [50] 2010	Caucasian	2624	510	14	8	902	42	2934	210	2	5099	378	7
Kato [48] 2009	African	68	43	4	70	41	4	–	–	–	–	–	–
Diergaarde [41] 2008	Mixed	258	61		495	142		291	26		584	49	
Singh [37] 2007	Indian	50	18	13	30	21	8	56	23	4	49	9	1
Li [32] 2006	Asian	–	–	–	–	–	–	31	44	14	35	74	27
Boyapati [24] 2005	Asian	147	157	55	165	182	70	429	273	34	441	273	44
Okobia [27] 2005	African	62	31	10	58	29	7	–	–	–	–	–	–
Chacko [29] 2005	Indian	33	25		46	12		36	22		45	13	
Li [22] 2004	Mixed	240	83	10	269	98	9	315	20	1	358	23	0
Zhang [21] 2004	Caucasian	211	78		209	52		255	34		248	13	
Laden [15] 2002	Mixed	237	56		239	54		250	43		258	35	
Huang [7] 1999	Asian	22	35	21	28	44	9	37	36	7	49	28	4
Moysich [10] 1999	Caucasian	–	–	–	–	–	–	127	27		168	23	
Ambrosone [2] 1995	Caucasian	–	–	–	–	–	–	140	32	4	195	31	2
Premenopausal													
Amrani [73] 2016	Asian	–	–	–	–	–	–	69	9		68	11	
Ghisari [67] 2014	Caucasian	–	–	–	–	–	–	2	14		17	25	
Martínez-Ramírez [64] 2013	Mixed	–	–	–	–	–	–	20	19	43	31	26	24
dos Santos [55] 2011	Mixed	16	14		36	3		–	–	–	–	–	–
Wang [54] 2011	Asian	–	–	–	–	–	–	120	84	20	124	84	16
Moreno-Galván [49] 2010	Mixed	8	15	4	15	24	5	10	16	1	13	22	9
Kato [48] 2009	African	49	25	5	39	26	9	–	–	–	–	–	–
Singh [37] 2007	Indian	40	17	5	57	21	3	39	16	5	51	29	1
Li [32] 2006	Asian	–	–	–	–	–	–	20	47	22	54	63	19
Boyapati [24] 2005	Asian	272	350	113	277	361	115	213	139	15	240	155	26
Okobia [27] 2005	African	78	40	7	71	43	10	–	–	–	–	–	–
Chacko [29] 2005	Indian	29	25		44	10		31	23		45	9	
Li [22] 2004	Mixed	242	88	15	221	87	11	329	21	2	294	25	2
Zhang [21] 2004	Caucasian	68	17		115	30		79	6		137	8	
Huang [7] 1999	Asian	27	25	11	20	36	8	34	28	1	31	25	8
