# Supplementary material for: Evaluation of association studies and a systematic review and meta-analysis of CYP1A1 T3801C and A2455G polymorphisms in breast cancer risk
Source: PLoS One. 2021 Apr 28;16(4):e0249632. doi: 10.1371/journal.pone.0249632 (PMC8081265; doi:10.1371/journal.pone.0249632)
Supplement: S4 Table — (PDF) [file pone.0249632.s004.pdf]

S4 Table Results of previous meta-analyses between CYP1A1 T3801C and A2455G polymorphisms with BC risk

First author/year	Variable	n (Cases/Controls)	CC vs. TT			TC vs. TT			CC vs. TT+ TC			TC + CC vs.TT			C vs T		
			OR (95% CI)	P_h/I^2 (%)	BFDP	OR (95% CI)	P_h/I^2 (%)	BFDP	OR (95% CI)	P_h/I^2 (%)	BFDP	OR (95% CI)	P_h/I^2 (%)	BFDP	OR (95% CI)	P_h/I^2 (%)	BFDP
Hussain [24] 2018	Overall	19	–	–	–	–	–	–	–	–	–	–	–	–	0.98 (0.68, 1.41)	<0.001/95.3	–
He [22] 2014	Overall	47 (16,272/20,930	1.23 (0.97, 1.56)	<0.001/72.6	–	1.06 (0.95, 1.18)	<0.001/65.7	–	1.18 (0.97, 1.45)	<0.001/66.4	–	1.07 (0.97, 1.19)	<0.001/72.9	–	–	<0.001/81.3	–
	Caucasian	15 (7467/11,710)	[illegible] 1.72)	0.041/47.3	–	1.05 (0.88, 1.26)	0.001/64.5	–	[illegible] 1.65)	0.057/44.1	–	1.05 (0.89, 1.23)	<0.001/67.9	–	[illegible] 1.31)	<0.001/75.6	–
	Asian	12 (3852/4045)	[illegible] 1.49)	<0.001/76.4	–	1.00 (0.83, 1.21)	0.010/61.9	–	[illegible] 1.38)	0.001/70.2	–	1.00 (0.83, 1.21)	<0.001/71.6	–	[illegible] 1.21)	<0.001/78.6	–
	African	5 (1011/1057)	[illegible] 2.25)	0.032/62.1	–	1.04 (0.87, 1.25)	0.177/36.7	–	[illegible] 2.03)	0.057/56.4	–	1.01 (0.76, 1.35)	0.085/51.2	–	[illegible] 1.35)	0.028/63.2	–
	South Indian	4 (863/782)	**[illegible] 6.03)**	0.208/34.0	**<0.001**	1.84 (1.11, 3.05)	0.004/77.1	0.996	[illegible] 3.49)	0.002/79.4	0.985	**3.40 (2.35, 4.94)**	0.473/0.0	**<0.001**	[illegible] 3.06)	0.002/79.1	0.926
	Mixed	8 (2608/2840)	[illegible] 2.04)	0.045/62.8	–	0.92 (0.80, 1.05)	0.178/39.0	–	[illegible] 1.29)	0.126/47.6	–	0.98 (0.81, 1.18)	0.096/42.4	–	[illegible] 1.27)	0.046/62.6	–
Sergentanis [19] 2010	Overall	32 (11909/16179)	0.95 (0.77, 1.17)	0.007	–	0.98 (0.88, 1.10)	0.002	–	0.95 (0.80, 1.13)	0.038	–	0.99 (0.90, 1.10)	<0.001	–	–	–	–
	Caucasian	15 (6598/10422)	0.89 (0.64, 1.23)	0.352	–	1.04 (0.85, 1.27)	0.008	–	0.89 (0.65, 1.23)	0.461	–	1.06 (0.91, 1.23)	0.005	–	–	–	–
	Chinese	9 (2981/3222)	0.95 (0.66, 1.37)	0.001	–	0.97 (0.76, 1.24)	0.006	–	0.96 (0.72, 1.28)	0.003	–	1.00 (0.79, 1.27)	<0.001	–	–	–	–
	African	5 (763/864)	1.09 (0.71, 1.65)	0.1	–	0.95 (0.77, 1.17)	0.285	–	1.10 (0.73, 1.66)	0.203	–	0.96 (0.78, 1.18)	0.124	–	–	–	–
Chen [17] 2007	Overall	13 (9316/12714)	1.09 (0.84, 1.40)	0.03	–	–	–	–	1.03 (0.89, 1.18)	0.18	–	0.95 (0.81, 1.11)	0.003	–	0.99 (0.87, 1.12)	0.001	–
	East Asian	4	1.17 (0.70, 1.97)	0.004	–	–	–	–	1.20 (0.81, 1.78)	0.02	–	1.00 (0.67, 1.50)	0.004	–	1.06 (0.80, 1.41)	0.0005	–
	Caucasian	3	1.08 (0.51, 2.31)	0.95	–	–	–	–	1.12 (0.52, 2.38)	0.96	–	0.83 (0.67, 1.03)	0.32	–	0.86 (0.71, 1.05)	0.32	–
	African	4	1.22 (0.76, 1.96)	0.11	–	–	–	–	1.24 (0.78, 1.96)	0.23	–	1.00 (0.66, 1.50)	0.07	–	1.02 (0.85, 1.24)	0.03	–
Wu [23] 2017	Chinese	4 (1605/1689)	1.08 (0.65, 1.78)	0.035	–	–	–	–	1.03 (0.82, 1.30)	0.35	–	1.01 (0.63, 1.60)	<0.001	–	1.01 (0.74, 1.37)	0.001	–
Ragin [18] 2010	African (USA)	4 (543/646)	1.19 (0.71, 1.99)	0.060/60	–	0.95 (0.74, 1.22)	0.170/40	–	–	–	–	–	–	–	–	–	–
	African	1 (220/218)	0.91 (0.41, 2.00)	–	–	–	–	–	–	–	–	–	–	–	–	–	–
Sengupta D [35] 2020	Indian	6 (NA)	–	–	–	–	–	–	2.23	0.35/32.45	0.982	2.20 (0.86–5.68)	0.004/82.0	–	–	–	–
**First author/year**	**Variable**	**n (Cases/Contro**	**TT versus CC**			**TC versus CC**			**TT + TC versus CC**			**TT versus TC + CC**			–	–	–
Yao L [20] 2010	Overall	23 (10520/14567)	0.93 (0.72, 1.19)	<0.001	–	0.95 (0.79, 1.14)	0.009	–	0.93 (0.75, 1.15)	<0.001	–	0.99 (0.87, 1.13)	<0.001	–	–	–	–
	Asian	10 (3748/3792)	0.85 (0.56, 1.27)	<0.001	–	0.88 (0.66, 1.17)	<0.001	–	0.85 (0.61, 1.20)	<0.001	–	0.95 (0.72, 1.24)	<0.001	–	–	–	–
	Caucasian	6 (4281/8152)	1.36 (0.88, 2.11)	0.881	–	1.37 (0.87, 2.14)	0.737	–	1.36 (0.88, 2.11)	0.868	–	1.06 (0.96, 1.17)	0.27	–	–	–	–
	African	5 (763/864)	0.88 (0.47, 1.65)	0.099	–	0.90 (0.59, 1.39)	0.503	–	0.93 (0.62, 1.40)	0.202	–	1.03 (0.85, 1.27)	0.124	–	–	–	–
	Mixed	2 (1728/1759)	1.13 (0.87, 1.47)	0.481	–	1.03 (0.79, 1.35)	0.575	–	1.09 (0.85, 1.41)	0.517	–	1.10 (0.96, 1.26)	0.643	–	–	–	–
**First author/year**	**Variable**	**n (Cases/Controls)**	**GG vs. AA**			**AG vs. AA**			**GG vs. AA+ AG**			**AG + GG vs. AA**			**G vs A**		
			**OR (95% CI)**	**P_h/I^2 (%)**	**BFDP**	**OR (95% CI)**	**P_h/I^2 (%)**	**BFDP**	**OR (95% CI)**	**P_h/I^2 (%)**	**BFDP**	**OR (95% CI)**	**P_h/I^2 (%)**	**BFDP**	**OR (95% CI)**	**P_h/I^2 (%)**	**BFDP**
Hussain T [24] 2018	Overall	15	–	–	–	–	–	–	–	–	–	–	–	–	0.89 (0.73, 1.09)	<0.001/73.3	
Qin J [21] 2014	Overall	38 (15969/24931)	1.05 (0.89, 1.24)	0.337/0.0	–	–	–	–	1.04 (0.88, 1.23)	0.416/3.3	–	1.00 (0.90, 1.10)	<0.001/54.7	–	–	–	–
	Caucasian	15 (9,117/17,585)	2.00 (1.05, 3.82)	0.987/0.0	0.997	–	–	–	2.00 (1.05, 3.82)	0.988/0.0	0.997	0.97 (0.83, 1.13)	0.068/38.0	–	–	–	–
	Asian	10 (3,760/4,342)	0.94 (0.78, 1.14)	0.388/5.7	–	–	–	–	0.94 (0.78, 1.13)	0.410/3.0	–	0.98 (0.89, 1.07)	0.309/14.5	–	–	–	–
	African	4 (829/872)	0.31 (0.01, 7.67)	–	–	–	–	–	0.31 (0.01, 7.60)	0.800/0.0	–	1.15 (0.69, 1.93)	0.800/0.0	–	–	–	–
	Indian	5 (952/816)	1.52 (0.48, 4.77)	0.047/58.5	–	–	–	–	1.62 (0.58, 4.50)	0.092/49.9	–	–	<0.001/90.8	–	–	–	–
	Mixed	4 (1,311/1,316)	1.45 (0.73, 2.87)	0.548/0.0	–	–	–	–	1.20 (0.64, 2.27)	0.350/0.0	–	1.06 (0.87, 1.30)	0.433/0.0	–	–	–	–
Sergentanis TN [19] 2010	Overall	29 (12257/20379)	1.14 (0.84, 1.54)	0.095	–	1.04 (0.91, 1.19)	0.001	–	0.98 (0.81, 1.20)	0.176	–	1.06 (0.93–1.20)	<0.001	–	–	–	–
	Caucasian	18 (9020/16462)	2.19 (1.25, 3.81)	0.161	0.99	1.06 (0.85, 1.32)	<0.001	–	2.08 (1.19, 3.61)	0.247	0.993	1.12 (0.91, 1.37)	<0.001	–	–	–	–
	Chinese	8 (2881/3458)	0.87 (0.70, 1.09)	0.578	–	1.00 (0.89, 1.12)	0.293	–	0.88 (0.71, 1.09)	0.617	–	0.95 (0.86, 1.06)	0.222	–	–	–	–
	African	3 (356/459)	1.69 (0.17, 16.4)	0.837	–	1.32 (0.64, 2.74)	0.778	–	1.66 (0.17, 16.1)	0.841	–	1.32 (0.64, 2.74)	0.778	–	–	–	–
Chen C [17] 2007	Overall	13 (9552/9320)	1.04 (0.61, 1.76)	0.08	–	–	–	–	0.85 (0.64, 1.12)	0.16	–	1.02 (0.82, 1.27)	0.005	–	1.01 (0.82, 1.25)	0.001	–
	East Asian	3	0.72 (0.53, 0.99)	0.95	0.998	–	–	–	0.73 (0.53, 0.99)	0.97	0.998	0.92 (0.68, 1.23)	0.09	–	0.91 (0.81, 1.03)	0.2	–
	Caucasian	5	2.11 (0.78, 5.75)	0.94	–	–	–	–	2.09 (0.77, 5.67)	0.95	–	0.95 (0.78, 1.17)	0.31	–	0.98 (0.81, 1.19)	0.29	–
	African	3	–	–	–	–	–	–	–	–	–	1.32 (0.64, 2.74)	0.78	–	1.31 (0.64, 2.70)	0.78	–
Wu H [23] 2017	Chinese	3 (1363/1490)	0.90 (0.56, 1.45)	0.204	–	–	–	–	0.82 (0.60, 1.11)	0.251	–	1.02 (0.87, 1.18)	0.343	–	1.00 (0.86, 1.17)	0.296	–
Ragin CC [18] 2010	African	4 (554/906)	–	–	–	–	–	–	–	–	–	0.83 (0.50, 1.37)	0.332/12	–	–	–	–
[illegible]	[illegible]	[illegible]							[illegible]	[illegible]		[illegible]	[illegible]				
