# Supplementary material for: Evaluation of association studies and a systematic review and meta-analysis of CYP1A1 T3801C and A2455G polymorphisms in breast cancer risk
Source: PLoS One. 2021 Apr 28;16(4):e0249632. doi: 10.1371/journal.pone.0249632 (PMC8081265; doi:10.1371/journal.pone.0249632)
Supplement: S1 Appendix — (DOCX) [file pone.0249632.s005.docx]

**Supplemental references**

1. Hayashi S, Watanabe J, Kawajiri K. High susceptibility to lung cancer analyzed in terms of combined genotypes of P450IA1 and Mu-class glutathione S-transferase genes. Jpn J Cancer Res. 1992;83:866–70. https://doi:org/10.1111/j.1349-7006.1992.tb01992.x
2. Ambrosone CB, Freudenheim JL, Graham S, Marshall JR, Vena JE, Brasure JR, Laughlin R, Nemoto T, Michalek AM, Harrington A, et al. Cytochrome P4501A1 and glutathione S-transferase (M1) genetic polymorphisms and postmenopausal breast cancer risk. Cancer Res. 1995;55:3483–5
3. Taioli E, Trachman J, Chen X, Toniolo P, Garte SJ. A CYP1A1 restriction fragment length polymorphism is associated with breast cancer in African-American women. Cancer Res. 1995;55:3757–8
4. Bailey LR, Roodi N, Verrier CS, Yee CJ, Dupont WD, Parl FF. Breast cancer and CYPIA1, GSTM1, and GSTT1 polymorphisms: evidence of a lack of association in Caucasians and African Americans. Cancer Res. 1998;58:65–70
5. Fontana X, Peyrottes I, Rossi C, Leblanc-Talent P, Ettore F, Namer M, Bussière F. Study of the frequencies of CYP1A1 gene polymorphisms and glutathione S-transferase mu1 gene in primary breast cancers: an update with an additional 114 cases. Mutat Res. 1998;403:45–53. https://doi:org/10.1016/s0027-5107(98)00025-6
6. Ishibe N, Hankinson SE, Colditz GA, Spiegelman D, Willett WC, Speizer FE, Kelsey KT, Hunter DJ. Cigarette smoking, cytochrome P450 1A1 polymorphisms, and breast cancer risk in the Nurses’ Health Study. Cancer Res. 1998;58:667–671. loss in tumor progression. Int J Gastrointest Cancer 2001;30:105–11
7. Huang CS, Shen CY, Chang KJ, Hsu SM, Chern HD. Cytochrome P4501A1 polymorphism as a susceptibility factor for breast cancer in postmenopausal Chinese women in Taiwan. Br J Cancer. 1999;80:1838–43. https://doi:org/10.1038/sj.bjc.6690608
8. Taioli E, Bradlow HL, Garbers SV, Sepkovic DW, Osborne MP, Trachman J, Ganguly S, Garte SJ. Role of estradiol metabolism and CYP1A1 polymorphisms in breast cancer risk. Cancer Detect Prev. 1999;23:232–7. https://doi:org/10.1046/j.1525-1500.1999.09912.x
9. Huang CS, Chern HD, Chang KJ, Cheng CW, Hsu SM, Shen CY. Breast cancer risk associated with genotype polymorphism of the estrogen-metabolizing genes CYP17, CYP1A1, and COMT: a multigenic study on cancer susceptibility. Cancer Res. 1999;59:4870–5
10. Moysich KB, Shields PG, Freudenheim JL, Schisterman EF, Vena JE, Kostyniak P, Greizerstein H, Marshall JR, Graham S, Ambrosone CB. Polychlorinated biphenyls, cytochrome P4501A1 polymorphism, and postmenopausal breast cancer risk. Cancer Epidemiol Biomarkers Prev. 1999;8:41–4
11. Basham VM, Pharoah PD, Healey CS, Luben RN, Day NE, Easton DF, Ponder BA, Dunning AM. Polymorphisms in CYP1A1 and smoking: no association with breast cancer risk. Carcinogenesis. 2001;22:1797–800. https://doi:org/10.1093/carcin/22.11.1797
12. Krajinovic M, Ghadirian P, Richer C, Sinnett H, Gandini S, Perret C, Lacroix A, Labuda D, Sinnett D. Genetic susceptibility to breast cancer in French-Canadians: role of carcinogen-metabolizing enzymes and gene-environment interactions. Int J Cancer. 2001;92:220–5. https://doi:org/10.1002/1097-0215(200102)9999:9999%3C::aid-ijc1184%3E3.0.co;2-h
13. Dialyna IA, Arvanitis DA, Spandidos DA. Genetic polymorphisms and transcriptional pattern analysis of CYP1A1, AhR, GSTM1, GSTP1 and GSTT1 genes in breast cancer. Int J Mol Med. 2001;8:79–87. https://doi:org/10.3892/ijmm.8.1.79
14. Miyoshi Y, Takahashi Y, Egawa C, Noguchi S. Breast cancer risk associated with CYP1A1 genetic polymorphisms in Japanese women. Breast J. 2002;8:209–15. https://doi:org/10.1046/j.1524-4741.2002.08404.x
15. Laden F, Ishibe N, Hankinson SE, Wolff MS, Gertig DM, Hunter DJ, Kelsey KT. Polychlorinated biphenyls, cytochrome P450 1A1, and breast cancer risk in the Nurses' Health Study. Cancer Epidemiol Biomarkers Prev. 2002;11:1560–5
16. Wu FY, Lee YJ, Chen DR, Kuo HW. Association of DNA-protein crosslinks and breast cancer. Mutat Res. 2002;501:69–78. https://doi:org/10.1016/s0027-5107(02)00006-4
17. da Fonte de Amorim L, Rossini A, Mendonça G, Lotsch P, de Almeida Simão T, de Moura Gallo C, Pinto L. CYP1A1, GSTM1, and GSTT1 polymorphisms and breast cancer risk in Brazilian women. Cancer Lett. 2002;181:179–86. https://doi:org/10.1016/s0304-3835(02)00058-7
18. Miyoshi Y, Ando A, Hasegawa S, Ishitobi M, Yamamura J, Irahara N, Tanji Y, Taguchi T, Tamaki Y, Noguchi S. Association of genetic polymorphisms in CYP19 and CYP1A1 with the oestrogen receptor-positive breast cancer risk. Eur J Cancer. 2003;39:2531–7. https://doi:org/10.1016/j.ejca.2003.08.017
19. Zhu J, Chang P, Bondy ML, Sahin AA, Singletary SE, Takahashi S, Shirai T, Li D. Detection of 2-amino-1-methyl-6-phenylimidazo[4,5-b]-pyridine-DNA adducts in normal breast tissues and risk of breast cancer. Cancer Epidemiol Biomarkers Prev. 2003;12:830–7
20. Hefler LA, Tempfer CB, Grimm C, Lebrecht A, Ulbrich E, Heinze G, Leodolter S, Schneeberger C, Mueller MW, Muendlein A, Koelbl H. Estrogen-metabolizing gene polymorphisms in the assessment of breast carcinoma risk and fibroadenoma risk in Caucasian women. Cancer. 2004;101:264–9. https://doi:org/10.1002/cncr.20361
21. Zhang Y, Wise JP, Holford TR, Xie H, Boyle P, Zahm SH, Rusiecki J, Zou K, Zhang B, Zhu Y, Owens PH, Zheng T. Serum polychlorinated biphenyls, cytochrome P-450 1A1 polymorphisms, and risk of breast cancer in Connecticut women. Am J Epidemiol. 2004;160:1177–83. https://doi:org/10.1093/aje/kwh346
22. Li Y, Millikan RC, Bell DA, Cui L, Tse CK, Newman B, Conway K. Cigarette smoking, cytochrome P4501A1 polymorphisms, and breast cancer among African-American and white women. Breast Cancer Res. 2004;6:R460–73. https://doi:org/10.1186/bcr814
23. Shen YP. Case-control study on the associations of genetic polymorphisms of CYP1A1 Msp I, ER PvuII and ER XbaI with the risk of breast cancer. PhD thesis of Fudan University. 2004; p1–122.
24. Boyapati SM, Shu XO, Gao YT, Cai Q, Jin F, Zheng W. Polymorphisms in CYP1A1 and breast carcinoma risk in a population-based case-control study of Chinese women. Cancer. 2005;103:2228–35. https://doi:org/10.1002/cncr.21056
25. Le Marchand L, Donlon T, Kolonel LN, Henderson BE, Wilkens LR. Estrogen metabolism-related genes and breast cancer risk: the multiethnic cohort study. Cancer Epidemiol Biomarkers Prev. 2005;14:1998–2003. https://doi:org/10.1158/1055-9965.EPI-05-0076
26. Li Y, Millikan RC, Bell DA, Cui L, Tse CK, Newman B, Conway K. Polychlorinated biphenyls, cytochrome P450 1A1 (*CYP1A1*) polymorphisms, and breast cancer risk among African American women and white women in North Carolina: a population-based case-control study. Breast Cancer Res. 2005;7:R12–8. https://doi:org/10.1186/bcr941
27. Okobia M, Bunker C, Zmuda J, Kammerer C, Vogel V, Uche E, Anyanwu S, Ezeome E, Ferrell R, Kuller L. Cytochrome P4501A1 genetic polymorphisms and breast cancer risk in Nigerian women. Breast Cancer Res Treat. 2005;94:285–93. https://doi:org/10.1007/s10549-005-9022-x
28. Modugno F, Zmuda JM, Potter D, Cai C, Ziv E, Cummings SR, Stone KL, Morin PA, Greene D, Cauley JA. Estrogen metabolizing polymorphisms and breast cancer risk among older white women. Breast Cancer Res Treat. 2005;93:261–70. https://doi:org/10.1007/s10549-005-5347-8
29. Chacko P, Joseph T, Mathew BS, Rajan B, Pillai MR. Role of xenobiotic metabolizing gene polymorphisms in breast cancer susceptibility and treatment outcome. Mutat Res. 2005;581:153–63. https://doi:org/10.1016/j.mrgentox.2004.11.018
30. Chang TW, Wang SM, Guo YL, Tsai PC, Huang CJ, Huang W. Glutathione S-transferase polymorphisms associated with risk of breast cancer in southern Taiwan. Breast. 2006;15:754–61. https://doi:org/10.1016/j.breast.2006.03.008
31. Gallicchio L, Berndt SI, McSorley MA, Newschaffer CJ, Thuita LW, Argani P, Hoffman SC, Helzlsouer KJ. Polymorphisms in estrogen-metabolizing and estrogen receptor genes and the risk of developing breast cancer among a cohort of women with benign breast disease. BMC Cancer. 2006;6:173. https://doi:org/10.1186/1471-2407-6-173
32. Li JY, Wu DS, Yang F, Zeng HY, Lei FM, Zhou WD, Li H, Tao P. Study on serum organochlorines pesticides (DDTs) level, CYP1A1 genetic polymorphism and risk of breast cancer: a case control study. Zhonghua Liu Xing Bing Xue Za Zhi. 2006;27:217–22.
33. Shen Y, Li DK, Wu J, Zhang Z, Gao E. Joint effects of the CYP1A1 MspI, ERalpha PvuII, and ERalpha XbaI polymorphisms on the risk of breast cancer: results from a population-based case-control study in Shanghai, China. Cancer Epidemiol Biomarkers Prev. 2006;15:342–7. https://doi:org/10.1158/1055-9965.EPI-05-0485
34. Singh V, Rastogi N, Sinha A, Kumar A, Mathur N, Singh MP. A study on the association of cytochrome-P450 1A1 polymorphism and breast cancer risk in north Indian women. Breast Cancer Res Treat. 2007;101:73–81. https://doi:org/10.1007/s10549-006-9264-2
35. Shin A, Kang D, Choi JY, Lee KM, Park SK, Noh DY, Ahn SH, Yoo KY. Cytochrome P450 1A1 (CYP1A1) polymorphisms and breast cancer risk in Korean women. Exp Mol Med. 2007;39:361–6. https://doi:org/10.1038/emm.2007.40
36. Sillanpää P, Heikinheimo L, Kataja V, Eskelinen M, Kosma VM, Uusitupa M, Vainio H, Metsola K, Hirvonen A. CYP1A1 and CYP1B1 genetic polymorphisms, smoking and breast cancer risk in a Finnish Caucasian population. Breast Cancer Res Treat. 2007;104:287–97. https://doi:org/10.1007/s10549-006-9414-6
37. Singh N, Mitra AK, Garg VK, Agarwal A, Sharma M, Chaturvedi R, Rath SK. Association of CYP1A1 polymorphisms with breast cancer in North Indian women. Oncol Res. 2007;16:587–97
38. Guo WD, Rao NL, Liu CL, Zhao W, Huo ZH, Peng L, Chen YT, Jiao HY. Study of CYP1A1 *2A Gene Polymorphism and Susceptibility to Breast Cancer in the Han Nationality in Ningxia. China Cancer. 2007;16: 686–8
39. Justenhoven C, Hamann U, Schubert F, Zapatka M, Pierl CB, Rabstein S, Selinski S, Mueller T, Ickstadt K, Gilbert M, et al. Breast cancer: a candidate gene approach across the estrogen metabolic pathway. Breast Cancer Res Treat. 2008;108:137–49. https://doi:org/10.1007/s10549-007-9586-8
40. Torresan C, Oliveira MM, Torrezan GT, de Oliveira SF, Abuázar CS, Losi-Guembarovski R, Lima RS, Urban CA, Cavalli IJ, Ribeiro EM. Genetic polymorphisms in oestrogen metabolic pathway and breast cancer: a positive association with combined CYP/GST genotypes. Clin Exp Med. 2008;8:65–71. https://doi:org/10.1007/s10238-008-0159-x
41. Diergaarde B, Potter JD, Jupe ER, Manjeshwar S, Shimasaki CD, Pugh TW, Defreese DC, Gramling BA, Evans I, White E. Polymorphisms in genes involved in sex hormone metabolism, estrogen plus progestin hormone therapy use, and risk of postmenopausal breast cancer. Cancer Epidemiol Biomarkers Prev. 2008;17:1751–9. https://doi:org/10.1158/1055-9965.EPI-08-0168
42. Li JY, Long QM, Tao P, Hu R, Li H, Lei FM, Zhou WD, Li SF. Using MSR model to analyze the impact of gene-gene interaction with related to the genetic polymorphism of metabolism enzymes on the risk of breast cancer. Sichuan Da Xue Xue Bao Yi Xue Ban. 2008;39:780–3, 7
43. Gulyaeva LF, Mikhailova ON, PustyInyak VO, Kim IV 4th, Gerasimov AV, Krasilnikov SE, Filipenko ML, Pechkovsky EV. Comparative analysis of SNP in estrogen-metabolizing enzymes for ovarian, endometrial, and breast cancers in Novosibirsk, Russia. Adv Exp Med Biol. 2008;617:359–66. https://doi:org/10.1007/978-0-387-69080-3_34
44. Ociepa-Zawal M, Rubiś B, Filas V, Breborowicz J, Trzeciak WH. Studies on CYP1A1, CYP1B1 and CYP3A4 gene polymorphisms in breast cancer patients. Ginekol Pol. 2009;80:819–23
45. Sangrajrang S, Sato Y, Sakamoto H, Ohnami S, Laird NM, Khuhaprema T, Brennan P, Boffetta P, Yoshida T. Genetic polymorphisms of estrogen metabolizing enzyme and breast cancer risk in Thai women. Int J Cancer. 2009;125:837–43. https://doi:org/10.1002/ijc.24434
46. Surekha D, Sailaja K, Rao DN, Padma T, Raghunadharao D, Vishnupriya S. Association of CYP1A1*2 polymorphisms with breast cancer risk: a case–control study. Indian J Med Sci. 2009;63:13–20. https://doi:org/10.4103/0976-9668.136150
47. Shimada N, Iwasaki M, Kasuga Y, Yokoyama S, Onuma H, Nishimura H, Kusama R, Hamada GS, Nishimoto IN, Iyeyasu H, et al. Genetic polymorphisms in estrogen metabolism and breast cancer risk in case–control studies in Japanese, Japanese Brazilians and non-Japanese Brazilians. J Hum Genet. 2009;54:209–15. https://doi.org/10.1038/jhg.2009.13
48. Kato I, Cichon M, Yee CL, Land S, Korczak JF. African American-preponderant single nucleotide polymorphisms (SNPs) and risk of breast cancer. Cancer Epidemiol. 2009;33:24–30. https://doi:org/10.1016/j.canep.2009.04.009
49. Moreno-Galván M, Herrera-González NE, Robles-Pérez V, Velasco-Rodríguez JC, Tapia-Conyer R, Sarti E. Impact of CYP1A1 and COMT genotypes on breast cancer risk in Mexican women: a pilot study. Int J Biol Markers. 2010;25:157–63
50. MARIE-GENICA Consortium on Genetic Susceptibility for Menopausal Hormone Therapy Related Breast Cancer Risk: Jenny Chang-Claude, Lars Beckmann, Charlotte Corson, Rebecca Hein, Silke Kropp, Margie Parthimos, Thomas Dünnebier, Ute Hamann, Benedikt Brors, Roland Eils, et al. Genetic polymorphisms in phase I and phase II enzymes and breast cancer risk associated with menopausal hormone therapy in postmenopausal women. Breast Cancer Res Treat. 2010;119:463–74. https://doi:org/10.1007/s10549-009-0407-0
51. Syamala VS, Syamala V, Sheeja VR, Kuttan R, Balakrishnan R, Ankathil R. Possible risk modification by polymorphisms of estrogen metabolizing genes in familial breast cancer susceptibility in an Indian population. Cancer Invest. 2010;28:304–11. https://doi:org/10.3109/07357900902744494
52. Cui Z, Qian BY, Chen KX, Zheng F, Ma J. The Relationship of CYP1A1, GSTT1 and GSTM1 with Susceptibility of Breast Cancer Based on a Case-Control Study. Chin J Prev Contr Chron Dis. 2010;18:248–52
53. Kiruthiga PV, Kannan MR, Saraswathi C, Pandian SK, Devi KP. CYP1A1 gene polymorphisms: lack of association with breast cancer susceptibility in the southern region (Madurai) of India. Asian Pac J Cancer Prev. 2011;2:2133–8
54. Wang Q, Li H, Tao P, Wang YP, Yuan P, Yang CX, Li JY, Yang F, Lee H, Huang Y. Soy isoflavones, CYP1A1, CYP1B1, and COMT polymorphisms, and breast cancer: a case-control study in southwestern China. DNA Cell Biol. 2011;30:585–95. https://doi:org/10.1089/dna.2010.1195
55. dos Santos RA, Teixeira AC, Mayorano MB, Carrara HH, de Andrade J, Takahashi CS. Variability in estrogen-metabolizing genes and their association with genomic instability in untreated breast cancer patients and healthy women. J Biomed Biotechnol. 2011;571784. https://doi:org/10.1155/2011/571784
56. Tuerxun M, Hamulati W, Bai L, Peng XM, Dolikun M. Study of cytochrome P450 1A1 gene 3'-UTR 6235T-C polymorphism and susceptibility to breast cancer with Uighur medicine. Zhonghua Yi Xue Za Zhi. 2011;91:86–91
57. Chen J. Study of CYP1A1 gene polymorphism and susceptibility to female breast cancer in the Han Nationality in Zunyi. Master's Degree Thesis of Zunyi Medical College. 2011;p1–50
58. Naushad SM, Reddy CA, Rupasree Y, Pavani A, Digumarti RR, Gottumukkala SR, Kuppusamy P, Kutala VK. Cross-talk between one-carbon metabolism and xenobiotic metabolism: implications on oxidative DNA damage and susceptibility to breast cancer. Cell Biochem Biophys. 2011;61:715–23. https://doi:org/10.1007/s12013-011-9245-x.
59. Ashley-Martin J, VanLeeuwen J, Cribb A, Andreou P, Guernsey JR. Breast cancer risk, fungicide exposure and CYP1A1*2A gene-environment interactions in a province-wide case control study in Prince Edward Island, Canada. Int J Environ Res Public Health. 2012;9:1846–58. https://doi:org/10.3390/ijerph9051846.
60. Reding KW, Chen C, Lowe K, Doody DR, Carlson CS, Chen CT, Houck J, Weiss LK, Marchbanks PA, Bernstein L, et al. Estrogen-related genes and their contribution to racial differences in breast cancer risk. Cancer Causes Control. 2012;23:671–81. https://doi:org/10.1007/s10552-012-9925-x.
61. Khvostova EP, Pustylnyak VO, Gulyaeva LF. Genetic polymorphism of estrogen metabolizing enzymes in Siberian women with breast cancer. Genet Test Mol Biomarkers. 2012;16:167–73. https://doi:org/10.1089/gtmb.2011.0131
62. Huang Y, Liu D, Rao SQ, Zhang HF. Association of CYP1A1 and CYP1A2 polymorphism with breast cancer in women of Han nationality. Chin J Breast Dis. 2013;7:345–50.
63. Wang H, Wang WJ. Relationship between CYP1A1 polymorphisms and invasion and metastasis of breast cancer. Asian Pac J Trop Med. 2013;6:835–8. https://doi:org/10.1016/S1995-7645(13)60148-0.
64. Martínez-Ramírez OC, Pérez-Morales R, Castro C, Flores-Díaz A, Soto-Cruz KE, Astorga-Ramos A, Gonsebatt ME, Casas L, Valdés-Flores M, Rubio J. Polymorphisms of catechol estrogens metabolism pathway genes and breast cancer risk in Mexican women. Breast. 2013;22:335–43. doi:org/10.1016/j.breast.2012.08.004.
65. Petchkovskiy EV, Shadrina AS, Boyarskih UA, Selezneva IA, Sinkina TV, Lazarev AF, Petrova VD, Filipenko ML. The polymorphism of genes of synthesis and metabolism of estrogens and the risk of breast cancer. Klin Lab Diagn. 2014;2:19–23.
66. Saadatian H, Gharesouran J, Montazeri V, Mohammadi SA, Mohaddes Ardabili SM. Polymorphism of the cytochrome P-450 1A1 (A2455G) in women with breast cancer in Eastern Azerbaijan, Iran. Iran J Basic Med Sci. 2014;17:227–30
67. Ghisari M, Eiberg H, Long M, Bonefeld-Jørgensen EC. Polymorphisms in phase I and phase II genes and breast cancer risk and relations to persistent organic pollutant exposure: a case-control study in Inuit women. Environ Health. 2014;13:19. doi:org/10.1186/1476-069X-13-19.
68. Zhang M, Gao CE, Chen Y, Chen D, Chen DD, Zou TN, Yang Y. Relationship between MspI polymorphism of CYP1A1 gene and susceptibility to breast cancer of Yi nationality in Yunnan. Med J West China. 2014;26:1581–4.
69. Zhang M, Gao GE, Chen Y, Chen DD, Zou TN, Li WH, Yang Y, Li Y. Relationship between Msp I polymorphism of CYP1A1 gene and susceptibility to breast cancer in Yi nationality in Yunnan province. The Journal of Practical Medicine. 2014;30:2398–401.
70. Oliveira CB, Cardoso-Filho C, Bossi LS, Lourenço GJ, Costa-Gurgel MS, Lima CS. Association of CYP1A1 A4889G and T6235C polymorphisms with the risk of sporadic breast cancer in Brazilian women. Clinics (Sao Paulo). 2015;70:680–5. https://doi:org/10.6061/clinics/2015(10)04
71. Tuerxun M, Hamulati W, Xia K, Peng XM, Ilham N, Dolikun M , Song MS. The Effect of Single and Combined Genotypes of CYP1A1, CYP1B1, NET and DAT1 on Breast Cancer Susceptibility in a Han Chinese Population. Life Science Research. 2015;19:5219
72. Zakiullah A, Khisroon M, Saeed M, Khan A, Khuda F, Ali al. Serum polychlorinated biphenyls, cytochrome P-450 1A1 polymorphisms, and risk of breast cancer in Connecticut women. Am J Epidemiol. 2004;160:1177–83
73. Amrani I, Bulatova N, Awidi A, Yousef AM, Melhem JM, Al-Masri M, Tahoun LA. Lack of Association between CYP1A1 M2 and M4 Polymorphisms and Breast Carcinoma in Jordanian Women: a Case–Control Study. Asian Pac J Cancer Prev. 2016;17:387–93. https://doi:org/10.7314/apjcp.2016.17.1.387
74. Farzaneh F, Noghabaei G, Barouti E, Pouresmaili F, Jamshidi J, Fazeli A, Emamalizadeh B, Darvish H. Analysis of CYP17, CYP19 and CYP1A1 Gene Polymorphisms in Iranian Women with Breast Cancer. Asian Pac J Cancer Prev. 2016;17:23–6. https://doi:org/10.7314/apjcp.2016.17.s3.23
75. Pazilat Abdurixit. CYP1A1 gene polymorphism and breast cancer correlation study of Uygur and Han. Master's Degree Thesis of Xinjiang Medical University.2016; p1–46.
76. García-Martínez A, Gamboa-Loira B, Tejero ME, Sierra-Santoyo A, Cebrián ME, López-Carrillo L. CYP1A1, CYP1B1, GSTM1 and GSTT1 genetic variants and breast cancer risk in Mexican women. Salud Publica Mex. 2017;59:540–7. https://doi:org/10.21149/8527.
77. Ghisari M, Long M, Røge DM, Olsen J, Bonefeld-Jørgensen EC. Polymorphism in xenobiotic and estrogen metabolizing genes, exposure to perfluorinated compounds and subsequent breast cancer risk: A nested case-control study in the Danish National Birth Cohort. Environ Res. 2017;154:325–33. https://doi:org/10.1016/j.envres.2017.01.020
78. Parada H Jr, Steck SE, Cleveland RJ, Teitelbaum SL, Neugut AI, Santella RM, Gammon MD. Genetic polymorphisms of phase I metabolizing enzyme genes, their interaction with lifetime grilled and smoked meat intake, and breast cancer incidence. Ann Epidemiol. 2017;27:208–14. https://doi:org/10.1016/j.annepidem.2016.11.005
79. Liang FQ, Zhang H, Du ZG, Zhou Y, Wan HY, Lv Q. Distribution of cytochrome P450 1A1 and 1B1 genes single nucleotide polymorphism on the breast cancer patients. Inter J Epidemiol Infect Dis. 2018;45:154–9.
80. Qiu J, Du Z, Liu J, Zhou Y, Liang F, Lü Q. Association between polymorphisms in estrogen metabolism genes and breast cancer development in Chinese women: A prospective case-control study. Medicine (Baltimore). 2018;97:e13337. https://doi:org/10.1097/MD.0000000000013337
81. Naif HM, Al-Obaide MAI, Hassani HH, Hamdan AS, Kalaf ZS. Association of Cytochrome CYP1A1 Gene Polymorphisms and Tobacco Smoking With the Risk of Breast Cancer in Women From Iraq. Front Public Health. 2018;6:96. https://doi:org/10.3389/fpubh.2018.00096
82. Khalili-Tanha G, Barzegar A, Nikbakhsh N, Ansari-Pirsaraei Z. Association of CYP1A1 M2 (A2455G) Polymorphism with Susceptibility to Breast Cancer in Mazandaran Province, Northern Iran: A Case-control Study. Int J Prev Med. 2019;10:92. https://doi:org/10.4103/ijpvm.IJPVM_57_18.
