# Supplementary material for: Evaluation of association studies and a systematic review and meta-analysis of CYP1A1 T3801C and A2455G polymorphisms in breast cancer risk
Source: PLoS One. 2021 Apr 28;16(4):e0249632. doi: 10.1371/journal.pone.0249632 (PMC8081265; doi:10.1371/journal.pone.0249632)
Supplement: S1 Table — (PDF) [file pone.0249632.s001.pdf]

S1 Table. Included studies of CYP1A1 polymorphisms in BC within the meta-analyses (A, Asian; Af, African; C, Caucasian; I, Indian; M, mixed; O, others).

| No. | First Author/Year | Country | Eligible research studies of rs4646903 (T3801C/MspI) | | | | | | | | | Eligible research studies of rs1048943 (A2455G/Ile462Val) | | | | | | | |
|---|---|---|---|---|---|---|---|---|---|---|---|---|---|---|---|---|---|---|---|
| | | | African | Indian | Chinese | All studies | | | | | | African | Indian | Chinese | All studies | | | | |
| | | | Ragin [18] 2010 | Sengupta [35] 2020 | Wu [23] 2017 | This study | Hussain [24] 2018 | He [22] 2014 | Yao [20] 2010 | Sergentanis [19] 2010 | Chen [17] 2007 | Ragin [18] 2010 | Sengupta [35] 2020 | Wu [23] 2017 | This study | Hussain [24] 2018 | Qin [21] 2014 | Sergentanis [19] 2010 | Chen [17] 2007 |
| 1 | Hayashi S [1] 1992 | Japan | – | – | – | – | – | – | – | – | – | – | – | – | A | – | A | Chinese | – |
| 2 | Ambrosone CB [2] 1995 | USA | – | – | – | – | – | – | – | – | – | – | – | – | C | – | C | C | C |
| 3 | Taioli E [3] 1995 | USA | – | – | – | Exclude | – | Exclude | – | – | Exclude | – | – | – | Exclude | – | Exclude | – | – |
| 4 | Bailey LR [4] 1998 | USA | Af | – | – | Af | USA | Af | Af | Af | Af | Af | – | – | Af | USA | Af | Af | Af |
| 5 | Bailey LR [4] 1998 | USA | – | – | – | C | | C | C | C | – | – | – | – | C | | C | C | C |
| 6 | Fontana X [5] 1998 | France | – | – | – | C | French | C | – | C | – | – | – | – | – | – | – | – | – |
| 7 | Ishibe N [6] 1998 | USA | – | – | – | M | – | M | – | C | – | – | – | – | M | – | M | C | – |
| 8 | Huang CS [7] 1999 | China | – | – | – | A | Chinese | A | A | Chinese | East Asian | – | – | – | A | Chinese | A | Chinese | East Asian |
| 9 | Taioli E [8] 1999 | USA | Af | – | – | C | USA | C | C | C | Af | Af | – | – | C | USA | C | C | C |
| 10 | Taioli E [8] 1999 | USA | | – | – | Af | | Af | Af | Af | | | – | – | Af | | Af | Af | Af |
| 11 | Huang CS [9] 1999 | China | – | – | – | Exclude | | Exclude | – | – | Exclude | – | – | Chinese | – | – | Exclude | – | – |
| 12 | Moysich KB [10] 1999 | USA | – | – | – | – | – | – | – | – | – | – | – | – | M | – | Exclude | – | – |
| 13 | Basham VM [11] 2001 | UK | – | – | – | – | – | – | – | – | – | – | – | – | C | UK | C | C | C |
| 14 | Krajinovic M [12] 2001 | Canada | – | – | – | C | French- | C | C | C | C | – | – | – | C | – | C | C | – |
| 15 | Dialyna IA [13] 2001 | Greek | – | – | – | C | – | C | – | C | – | – | – | – | – | – | – | – | – |
| 16 | Miyoshi Y [14] 2002 | Japan | – | – | – | A | Japanese | A | A | Chinese | East Asian | – | – | – | A | Japanese | A | Chinese | East Asian |
| 17 | Laden F [15] 2002 | USA | – | – | – | M | – | M | – | – | – | – | – | – | M | – | M | – | – |
| 18 | Wu FY [16] 2002 | China | – | – | – | A | – | A | – | Chinese | – | – | – | – | – | – | – | – | – |
| 19 | da Fonte de Amorim L [17] 200 | Brazil | – | – | – | C | – | M | – | M | – | – | – | – | – | – | – | – | – |
| 20 | da Fonte de Amorim L [17] 200 | Brazil | – | – | – | M | – | | – | | – | – | – | – | – | – | – | – | – |
| 21 | Miyoshi Y [18] 2003 | Japan | – | – | – | A | – | A | – | – | – | – | – | – | – | – | – | – | – |
| 22 | Zhu J [19] 2003 | USA | – | – | – | M | – | M | – | M | – | – | – | – | – | – | – | – | – |
| 23 | Hefler LA [20] 2004 | Germany | – | – | – | C | Australia | C | C | C | C | – | – | – | C | – | C | C | – |
| 24 | Zhang Y [21] 2004 | USA | – | – | – | C | USA | C | – | C | – | – | – | – | C | USA | C | C | – |
| 25 | Li Y [22] 2004 | USA | – | – | – | C | USA | C | C | C | C | – | – | – | C | USA | C | C | C |
| 26 | Li Y [22] 2004 | USA | Af | – | – | Af | | Af | Af | Af | Af | Af | – | – | Af | | Af | Af | Af |
| 27 | Shen YP [23] 2004 | China | – | – | – | Exclude | – | – | – | – | – | – | – | – | – | – | – | – | – |
| 28 | Boyapati SM [24] 2005 | China | – | – | Chinese | A | USA | A | A | Chinese | East Asian | – | – | Chinese | A | Chinese | A | Chinese | East Asian |
| 29 | Le Marchand L [25] 2005 | USA | – | – | – | M | USA | M | M | M | M (O) | – | – | – | – | – | – | – | – |
| 30 | Li Y [26] 2005 | China | – | – | – | Exclude | – | Exclude | – | – | – | – | – | – | – | – | Exclude | – | – |
| 31 | Okobia M [27] 2005 | Nigeria | Af | – | – | Af | Nigerians | Af | Af | Af | Af | – | – | – | – | – | – | – | – |
| 32 | Modugno F [28] 2005 | USA | – | – | – | – | – | – | – | – | – | – | – | – | C | – | C | C | – |
| 33 | Chacko P [29] 2005 | India | – | I | – | I | I | I | – | C | – | – | I | – | I | I | I | C | I (O) |
| 34 | Chang TW [30] 2006 | China | – | – | – | A | – | A | – | Chinese | – | – | – | – | A | – | A | Chinese | – |
| 35 | Gallicchio L [31] 2006 | USA | – | – | – | – | – | – | – | – | – | – | – | – | C | – | C | – | – |
| 36 | Li JY [32] 2006 | China | – | – | – | – | – | – | – | – | – | – | – | Chinese | A | – | A | Chinese | – |
| 37 | Shen Y [33] 2006 | China | – | – | Chinese | A | Chinese | A | A | Chinese | East Asian | – | – | – | – | – | – | – | – |
| 38 | Singh V [34] 2007 | India | – | I | – | Exclude | I | I | A | C | I (O) | – | I | – | – | I | I | C | I (O) |
| 39 | Shin A [35] 2007 | Korea | – | – | – | A | – | A (not in | A | Chinese | – | – | – | – | A | – | A | Chinese | – |
| 40 | Sillanpää P [36] 2007 | Filand | – | – | – | – | – | – | – | – | – | – | – | – | C | – | C | C | – |
| 41 | Singh N [37] 2007 | India | – | I | – | I | – | I | A | C | – | – | I | – | I | – | I | C | – |
| 42 | Guo WD [38] 2007 | China | – | – | – | A | – | – | – | – | – | – | – | – | – | – | – | – | – |
| 43 | Justenhoven C [39] 2008 | UK | – | – | – | Exclude | – | Exclude | – | – | – | – | – | – | Exclude | – | Exclude | – | – |
| 44 | Torresan C [40] 2008 | Brazil | – | – | – | M | – | M | – | C | – | – | – | – | – | – | – | – | – |
| 45 | Diergaarde B [41] 2008 | USA | – | – | – | M | – | C | – | C | – | – | – | – | M | – | C | C | – |
| 46 | Li JY [42] 2008 | China | – | – | – | A | – | A | – | Chinese | – | – | – | – | – | – | – | – | – |
| 47 | Gulyaeva LF [43] 2008 | Russia | – | – | – | C | – | C | – | – | – | – | – | – | – | – | – | C | – |
| 48 | Ociepa-Zawal M [44] 2009 | Poland | – | – | – | C | – | C | – | – | – | – | – | – | C | – | C | – | – |
| 49 | Sangrajrang S [45] 2009 | Thailand | – | – | – | A | – | A | A | Chinese | – | – | – | – | A | – | A | Chinese | – |
| 50 | Surekha D [46] 2009 | India | – | – | – | – | – | – | – | – | – | – | I | – | I | – | (not in HW | C | – |
| 51 | Shimada N [47] 2009 | Brazil | – | – | – | A (Japan) | – | A | A | – | – | – | – | – | A (Japan) | – | A | – | – |
| 52 | Shimada N [47] 2009 | Brazil | – | – | – | M | – | M | M | – | – | – | – | – | M | – | M | – | – |
| 53 | Shimada N [47] 2009 | Brazil | – | – | – | A (O) | – | – | – | – | – | – | – | – | A (O) | | | | |
| 54 | Kato I [48] 2009 | USA | Af | – | – | Af | – | Af | Af | Af | – | Af | – | – | – | – | – | – | – |
| 55 | Moreno-Galván M [49] 2010 | Mexico | – | – | – | M | – | M | – | – | – | – | – | – | M | – | M | – | – |
| 56 | MARIE-GENICA [50] 2010 | Germany | – | – | – | C | – | C | C | C | – | – | – | – | C | – | C | C | – |
| 57 | Syamala VS [51] 2010 | India | – | I | – | I | – | I | A | – | – | – | – | – | – | – | – | – | – |
| 58 | Cui Z [52] 2010 | China | – | – | – | A | – | – | – | – | – | – | – | – | – | – | – | – | – |
| 59 | Kiruthiga PV [53] 2011 | India | – | I | – | I | I | I | – | – | – | – | I | – | I | I | – | – | – |
| 60 | Wang Q [54] 2011 | China | – | – | – | – | – | – | – | – | – | – | – | – | A | – | A | – | – |
| 61 | dos Santos RA [55] 2011 | Brazil | – | – | – | M | – | – | – | – | – | – | – | – | – | – | – | – | – |
| 62 | Tuerxun M [56] 2011 | C | – | – | – | A | – | A | – | – | – | – | – | – | – | – | – | – | – |
| 63 | Chen J [57] 2011 | C | – | – | – | A | – | – | – | – | – | – | – | – | – | – | – | – | – |
| 64 | Naushad SM [58] 2011 | India | – | I | – | I | I | I | – | – | – | – | I | – | I | I | I | – | – |
| 65 | Ashley-Martin J [59] 2012 | Canada | – | – | – | C | – | C | – | – | – | – | – | – | – | – | – | – | – |
| 66 | Reding KW [60] 2012 | USA | – | – | – | C | – | C | – | – | – | – | – | – | C | – | C | – | – |
| 67 | Reding KW [60] 2012 | USA | | – | – | Af | – | Af | – | – | – | – | – | – | Af | – | Af | – | – |
| 68 | Khvostova EP [61] 2012 | Russia | – | – | – | C | – | C | – | – | – | – | – | – | – | – | – | – | – |
| 69 | Huang Y [62] 2013 | China | – | – | Chinese | A | – | – | – | – | – | – | – | – | – | – | – | – | – |
| 70 | Wang H [63] 2013 | China | – | – | Chinese | A | – | – | – | – | – | – | – | – | – | – | – | – | – |
| 71 | Martínez-Ramírez OC [64] 201 | Mexico | – | – | – | – | – | – | – | – | – | – | – | – | M | – | – | – | – |
| 72 | Petchkovskiy EV [65] 2014 | Russia | – | – | – | – | – | – | – | – | – | – | – | – | C | – | – | – | – |
| 73 | Saadatian H [66] 2014 | Iran | – | – | – | – | – | – | – | – | – | – | – | – | C | – | – | – | – |
| 74 | Ghisari M [67] 2014 | Danish | – | – | – | – | – | – | – | – | – | – | – | – | C | – | – | – | – |
| 75 | Zhang M [68] 2014 | China | – | – | – | A | – | – | – | – | – | – | – | – | – | – | – | – | – |
| 76 | Zhang M [69] 2014 | China | – | – | – | Exclude | – | – | – | – | – | – | – | – | – | – | – | – | – |
| 77 | Oliveira CB [70] 2015 | Brazil | – | – | – | M | – | – | – | – | – | – | – | – | M | – | – | – | – |
| 78 | Tuerxun M [71] 2015 | China | – | – | – | A | – | – | – | – | – | – | – | – | – | – | – | – | – |
| 79 | Zakiullah A [72] 2015 | – | – | – | – | Exclude | Pakistanis | – | – | – | – | – | – | – | – | – | – | – | – |
| 80 | Amrani I [73] 2016 | Jordan | – | – | – | – | – | – | – | – | – | – | – | – | C | Jordanian | – | – | – |
| 81 | Farzaneh F [74] 2016 | Iran | – | – | – | C | – | – | – | – | – | – | – | – | – | – | – | – | – |
| 82 | Pazilat Abdurixit [75] 2016 | China | – | – | – | A | – | – | – | – | – | – | – | – | – | – | – | – | – |
| 83 | García-Martínez A [76] 2017 | Mexico | – | – | – | M | – | – | – | – | – | – | – | – | M | – | – | – | – |
| 84 | Ghisari M [77] 2017 | Danish | – | – | – | – | – | – | – | – | – | – | – | – | C | – | – | – | – |
| 85 | Parada H [78] 2017 | USA | – | – | – | – | – | – | – | – | – | – | – | – | C | USA | – | – | – |
| 86 | Liang FQ [79] 2018 | China | – | – | – | A | – | – | – | – | – | – | – | – | – | – | – | – | – |
| 87 | Qiu J [80] 2018 | China | – | – | – | A | – | – | – | – | – | – | – | – | – | – | – | – | – |
| 88 | Naif HM [81] 2018 | Iraq | – | – | – | C | – | – | – | – | – | – | – | – | C | – | – | – | – |
